# Impact of partial bile duct ligation with or without repeated magnetic resonance imaging examinations in mice

**DOI:** 10.1038/s41598-022-25318-8

**Published:** 2022-12-05

**Authors:** Taili Chen, Zi-Yi Zhou, Jia-Yi Liu, Li-Yun Zheng, Zi-Wei Wang, Xiao-Jie Zhang, Shan Zeng

**Affiliations:** 1 Department of Oncology, Xiangya Hospital, Central South University, Changsha, 410011 Hunan Province China; 2 Department of Radiology, The Second Xiangya Hospital, Central South University, Changsha, 410011 Hunan Province China; 3 MR Collaboration, Central Research Institute, United Imaging Healthcare, Shanghai, 201800 China; 4 Department of Psychiatry, The Second Xiangya Hospital, Central South University, Changsha, 410011 Hunan Province China

**Keywords:** Gastrointestinal diseases, Biological techniques, Chemical biology, Physiology, Psychology, Zoology

## Abstract

Partial bile duct ligation (pBDL) is considered a well-tolerated cholestatic model. Magnetic resonance imaging (MRI) is one of the most widely used tools in noninvasive imaging. However, no systematic studies have reported the possible effects of repeated MRI assessments in the pBDL model. Sixty BALB/C mice were investigated. MRI images of each mouse were recorded once every 2 weeks for 6 weeks after pBDL or sham surgery. The reproducibility of the pBDL model and the reliability of MRI were examined by behavioral, physiological, biochemical, and pathological parameters. The mice showed no alterations on behavioral and physiological tests (*P* > 0.05) at 2, 4, and 6 weeks after pBDL. Repeated general anesthesia did not result in any impairment after pBDL (*P* > 0.05). The behavioral and biochemical parameters were not affected by repeated MRIs or repeated contrast-enhanced MRIs (*P* > 0.05). Pathological staining showed the homogeneous formation of collagenous fiber in the pBDL mice and did not indicate any influence of repeated contrast-enhanced MRI on the number of inflammatory cells or fibrotic formation (*P* > 0.05). Thus, pBDL is a reproducible model with many advantages for animal welfare and scientific research. Additionally, MRI, as a safe tool for longitudinal evaluation and is well tolerated in mice with cholestasis.

## Introduction

Cholestasis is defined as a decrease in bile flow because of an obstruction in the flow of bile or its impaired secretion by hepatocytes. It can cause the accumulation of bile, which leads to liver tissue damage, inflammation, and ultimately biliary fibrosis^1^. Bile duct ligation (BDL) has been widely used for decades as an experimental model for cholestatic liver fibrosis. However, because the ligation of the common bile duct results in higher mortality^2–4^, more animals are required than expected. Partial bile duct ligation (pBDL) offers several advantages over traditional BDL^5^. First, in the unligated lobes, normal liver tissue with a complete Glisson system can help preserve liver function. Second, the unligated lobes act as ideally matched controls for the ligated left lobes under the same systemic effects^6,7^. Hence, pBDL can directly distinguish the differences between the ligated and unligated lobes and reduce the number of animals needed. Nevertheless, concerning animal welfare, the effect of pBDL is not known to decrease the relevant complications of cholestasis, such as behavioral disorders, or abnormal mental states. Moreover, no relevant studies have been published to show intrahepatic differences in pBDL in vivo.

Magnetic resonance imaging (MRI) is a widely used non-invasive technology that has been employed for the diagnosis and grading of hepatic fibrosis in clinical practice^8^. In preclinical studies, MRI has been shown to differentiate pathological changes related to pBDL^9^. Although MRI is well tolerated in humans, a few important differences need to be highlighted in animal studies^10^. To begin with, animals need to be under general anesthesia and tightly bound in an animal fixator during the imaging. Animal handling may easily cause anxiety and stress responses^11^. Additionally, while general anesthetics are known to act at some sites within the central nervous system, the influences of animal behavior on general anesthesia are not fully understood^12^. Furthermore, because of the long duration of the MRI procedure, inadequate anesthesia is inevitable; thus, the high noise levels of the MRI apparatus during scanning can also cause physiological changes in animals^13^. Additionally, for each experimental mouse, repeated MRI examinations and intravenous injection of contrast agents may increase the incidence of the potential risks mentioned above.

The objective of this study was to systematically understand the potential negative effects of repeated MRI examinations in the pBDL models. First, we evaluated the adverse effects of the pBDL model compared to sham surgery. Then, we administered the MRI protocols of the pBDL model with a higher decibel of noise and longer scan time, which helped us find the possible effects of MRI. Finally, different time points were set up to evaluate the long-term influence of several experimental conditions.

## Methods

The experimental protocol (No. 2020494) was approved by the animal care committee of the Second Xiangya Hospital of Central South University. All animal studies were performed according to the institutional guidelines and complied with the ARRIVE guidelines.

### Animal and experimental groups

**Figure 1:**
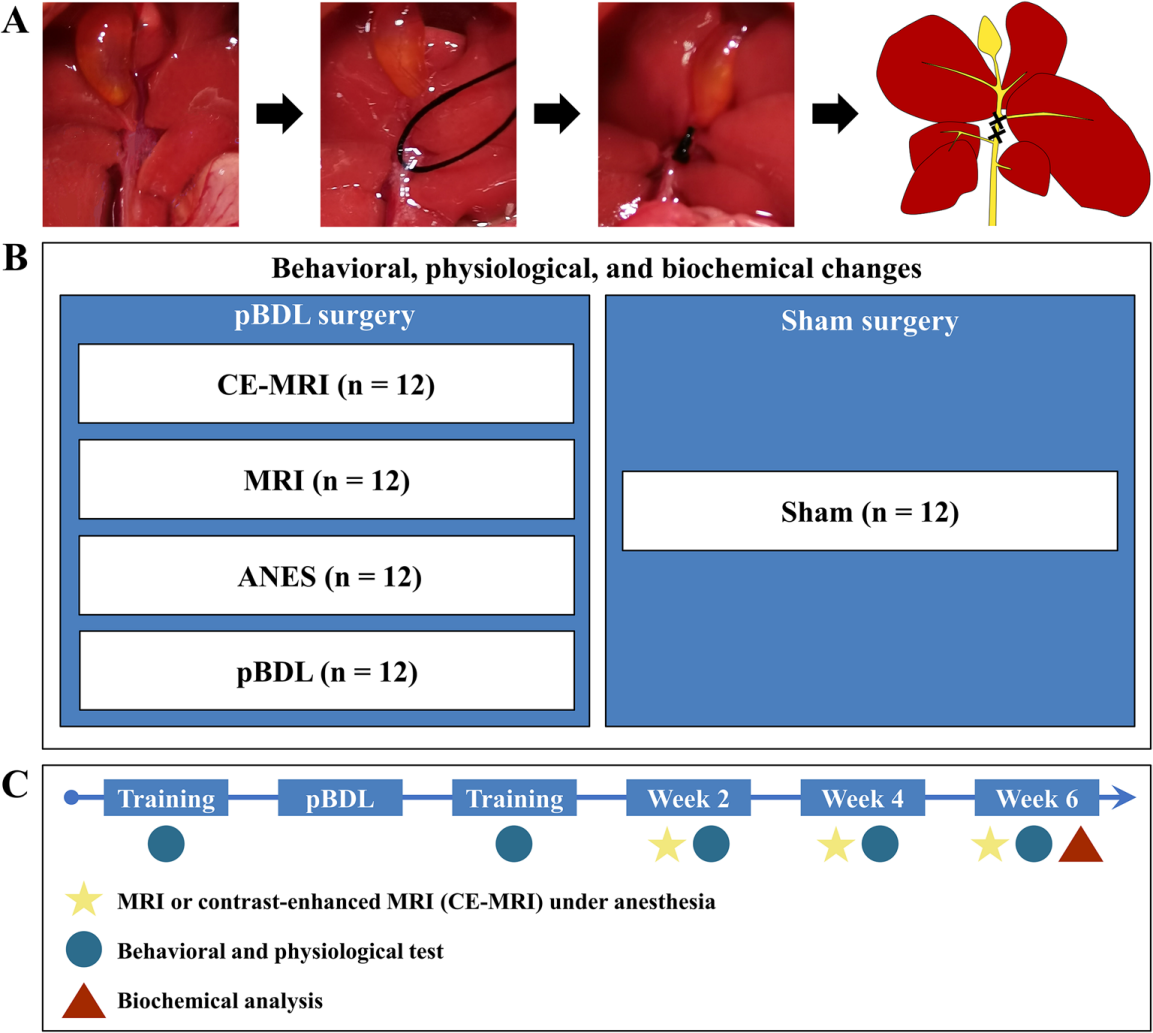
(**A**) Surgical photographs and ligated location demonstrating the technique for pBDL. (**B**) Overview of the experimental groups. (**C**) Timeline of the animal experimental procedure. Sham, only suture the abdominal wall. pBDL, partial bile duct ligation surgery alone; ANES, repeated anesthesia in the pBDL model; MRI, repeated magnetic resonance imaging under anesthesia in the pBDL model; CE-MRI, repeated contrast-enhanced magnetic resonance imaging under anesthesia in the pBDL model.

Based on the maximum feeding capacity of our animal laboratory, 60 male or female BALB/C mice aged 8 ~ 10 weeks were acquired from the Shanghai Laboratory Animal Center, Chinese Academy of Sciences for the experiments. Five groups (n = 12) including one sham and four pBDL groups (Fig. 1A) were randomly established via a random team generator. The details (Fig. 1B) for the four pBDL groups as follows: (1) pBDL surgery alone (pBDL); (2) pBDL surgery + repeated anesthesia (ANES); (3) pBDL surgery + repeated MRI with anesthesia (MRI); (4) pBDL surgery + repeated contrast enhanced MRI with anesthesia (CE-MRI). All animals were housed under optimal laboratory conditions (20–26℃ in a regular light–dark cycle). Purified water and standard pellet chow (SJA company, Hunan, China) were offered ad libitum.

### Surgical procedures

After fasting for 24 h, the mice were anesthetized by an intraperitoneal injection of 50 mg/kg pentobarbital sodium solution and 0.5 mg/kg medetomidine. Then, the abdominal fur of the mice was shaved and their legs were fixed the with medical tape. For the sham surgery, the abdomen was opened with a midline laparotomy about 2 cm in length and the abdominal wall was sutured subsequently. For the pBDL surgery, after opening the abdomen, a moistened cotton swab was used to gently retract the liver toward the head of the mouse. After confirming the anatomy of the hepatic bile duct and identifying the confluence of the left and the median bile duct, a 5–0 suture was placed around the confluence and secured with two surgical knots, while taking care to avoid injuring the hepatic artery and the liver surface (Fig. 1A). Finally, the abdominal layers were closed with 6–0 absorbable tape. After the surgery, 0.1 ml of gentamycin (200 U/ml) was injected subcutaneously, followed by a consecutive injection every 24 h for 48 h.

### MRI protocol

The MR examination was performed using a 3.0-Tesla clinical MRI (uMR 790, United Imaging Healthcare, Shanghai, China) with an 8-channel mouse coil. For each mouse in the MRI and CE-MRI groups, images were recorded once every 2 weeks for 6 weeks, starting at week 2 after the pBDL surgery (Fig. 1C). The mice were anesthetized with 50 mg/kg pentobarbital sodium solution by intraperitoneal injection before the MRI procedure. The abdomen of each mouse was fixed with medical tape to restrict respiratory movement.

The MRI protocol included one localization sequence, two routine sequences, and five functional sequences with higher decibel of noise and longer scan time. The following sequences and parameters were used: (1) transverse T1-weighted fast spin echo (FSE) sequence (repetition time (TR)/echo time (TE) = 585.0/12.6 ms, field of view (FOV) = 35 × 40 mm^2^, flip angle (FA) = 150°, slice thickness = 1.2 mm, matrix = 196 × 224); (2) transverse T2-weighted FSE sequence (TR/TE = 3243.0/85.9 ms, FOV = 40 × 40 mm^2^, FA = 140°, slice thickness = 1.5 mm, matrix = 224 × 224); (3) intravoxel incoherent motion imaging (IVIM, TR/TE = 585.0/12.6 ms, FOV = 35 × 40 mm^2^, FA = 150°, slice thickness = 1.2 mm, matrix = 64 × 64, b-values = 0, 50, 100, 150, 200, 400, 600, 800, 1000 and 1200 s/mm^2^); (4) T1 mapping (TR/TE = 15.0/3.9 ms, FOV = 75 × 75 mm^2^, FA = 5° and 26°, slice thickness = 1 mm, matrix = 320 × 320); (5) T2 mapping (TR = 694.7 ms, TE = 6.7, 17.9, 29.1, 40.3, and 51.4 ms, FOV = 90 × 90 mm^2^, FA = 60°, slice thickness = 2 mm, matrix = 320 × 320); (6) Magnetic resonance cholangiopancreatography (MRCP, TR/TE = 4500/298 ms, FOV = 110 × 110 mm^2^, FA = 160°, slice thickness = 20 mm, matrix = 270 × 270); (7) Dynamic contrast-enhanced imaging (DCE-MRI) immediately after a retro-orbital injection of a 0.1 ml solution comprising 0.02 ml gadobenate dimeglumine (Bracco Sine, Shanghai, China) and 0.08 ml normal saline for Gd-enhancement. Thirty measurements were acquired with DCE-MRI with each lasting 12.8 s. The total examination time for the whole MRI protocol was 28 min 50 s.

### Behavioral and physiological test

All animals were trained for behavioral and physiological examinations on preoperative day 3 and postoperative day 3. Thereafter, the data from the behavioral and physiological tests were recorded on the day after each intervention (Fig. 1C). The rotarod test was applied to discover the differences in motor coordination and balance among groups. The mice were placed on a cylinder with a circumference of 13 cm and allowed to adapt to the testing environment for a period of 15 s. The speed of rotation of the cylinder was gradually increased from 0 to a maximum of 40 rpm in 2 min and was stopped at 5 min if the mice managed to stay on for this duration of time. The speed when the mouse fell off the spinning cylinder and the latency to fall was recorded for each trial. An open field test was performed to measure the locomotor activity and anxiety-like behavior. We used a square open field box (34 cm × 34 cm × 34 cm) for the test. Each mouse was placed in the center of the box and allowed to move freely under 100 lx for 5 min. A digital camera positioned over the top of the box and electronic software was used for tracking. The total distance moved, average velocity, and the time spent at the central zone were recorded automatically.

To evaluate the physiological changes due to pBDL with or without repeated MRI, the heart rate and blood pressure were tested to determine whether well-documented inconsistencies existed between different groups. After the behavioral tests, all mice were transferred to a physiology laboratory and allowed to get acclimatized to the new surroundings for at least 1 h. The conscious mice were fixed, and the tail was placed inside a tail-cuff sensor. Automated noninvasive plethysmography was used to determine the blood pressure and heart rate.

### Biochemical and histopathological examination

The biochemical method was used to observe the changes in vivo between groups (Fig. 1C). After a final assessment via the behavioral tests in the last week, blood from the mice was harvested from the retro-orbital venous plexus and plasma was obtained by centrifugation at 3000 rpm for 10 min and stored at -80 °C until the further tests. The levels of alanine transaminase (ALT), aspartate transaminase (AST), alkaline phosphatase (ALP), albumin (ALB), creatinine (Crea), and urea were analyzed by a fully automatic biochemical analysis meter (chemray 240, Rayto, China) according to the manufacturer’s instructions.

All mice were killed after blood collection. To further confirm the morphologic and fibrotic changes in liver tissues, such as inflammatory formation and intrahepatic collagen deposition, paired liver samples of each mouse (right lobe and left-median lobe) were fixed using 4% paraformaldehyde, followed by dehydration and embedding in paraffin. Then, the embedded tissues were stained with hematoxylin and eosin (HE) and a commercial kit of Masson’s trichrome (G1006, Servicebio, China) according to the manufacturer’s protocol. The histological picture of each section was captured using a light microscope (Eclipse e100, Nikon, Japan) with a high-resolution camera (DS-U3, Nikon, Japan) and quantified using the ImageJ software (Rawak Software, Stuttgart, Germany). All steps were done independently by the assigned researchers. None of them knew about the results obtained by other researchers.

### Statistical analysis

Statistical analysis was performed with SPSS software 22.0 (IBM Corporation, Chicago, USA), and visualized by GraphPad prism 7.0 (GraphPad Software, California, USA). Comparisons between groups were performed by analysis of variance, student’s t-test, or the Mann–Whitney U test. For all analyses, a P value < 0.05 was considered statistically significant.

## Results

### Impact of the pBDL model on animal behavior, physiology, and biochemistry

**Figure 2:**
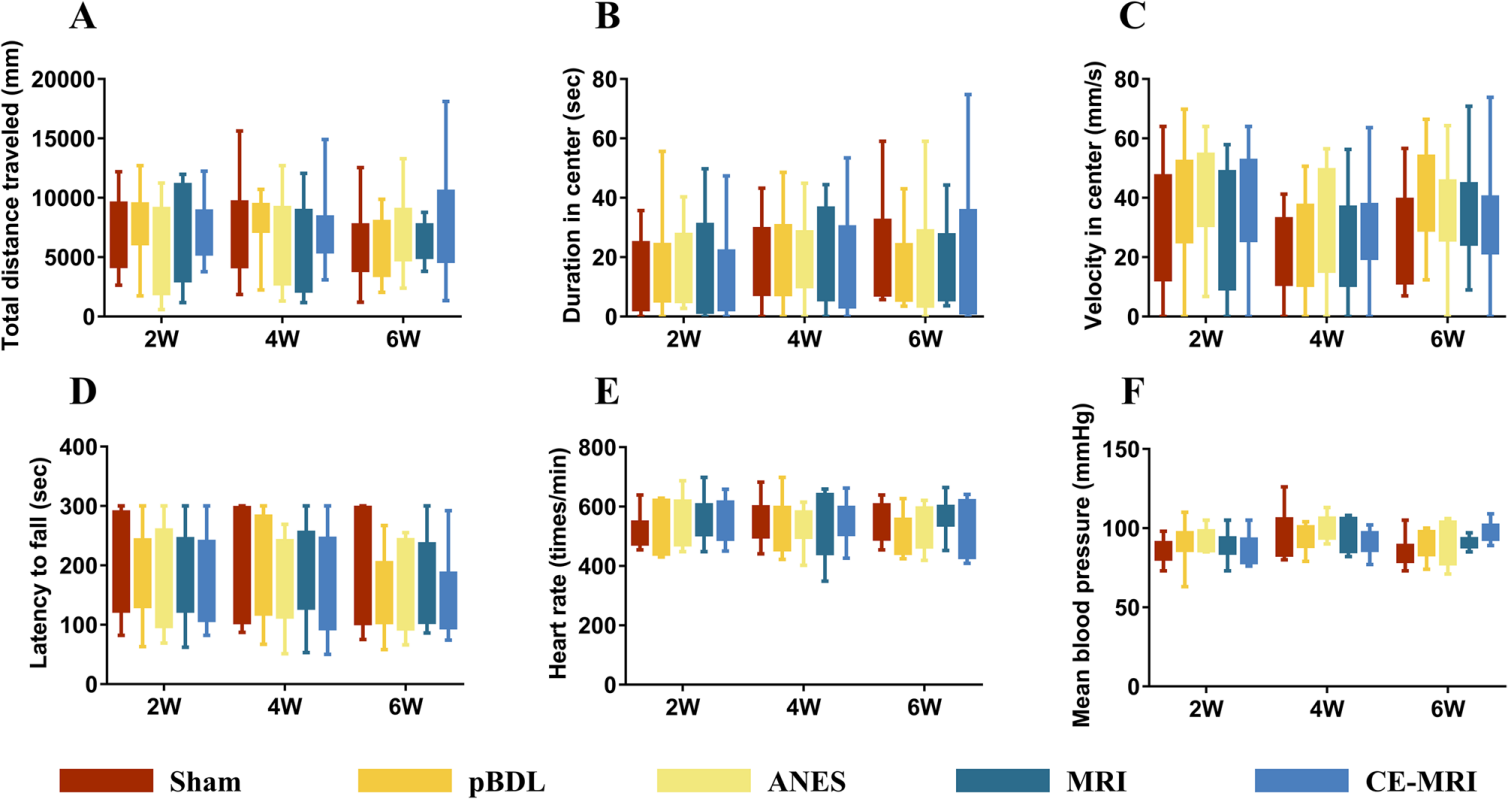
Behavioral and physiological data on weeks 2, 4, and 6 in all groups. (**A**–**D**) No differences were found in the open-field and rotarod test between the sham group and the remaining experimental groups. (**E**) and (**F**). No obvious changes in the heart rate and mean blood pressure parameters among the different groups. Sham, only suture the abdominal wall. pBDL, partial bile duct ligation surgery alone; ANES, repeated anesthesia in the pBDL model; MRI, repeated magnetic resonance imaging under anesthesia in the pBDL model; CE-MRI, repeated contrast-enhanced magnetic resonance imaging under anesthesia in the pBDL model.

The comparison showed that pBDL mice did not move a farther distance than the sham mice in the open field test (Fig. 2A). The pBDL mice did not spend more time or run faster to avoid the center of the arena than the sham mice (Fig. 2B,C). To verify that there were no motor balance impairments, mice were tested on the rotarod. A shorter latency to fall was related to impaired motor coordination. The groups showed no significant differences across the tests (Fig. 2D), indicating normal motor balance.

**Figure 3:**
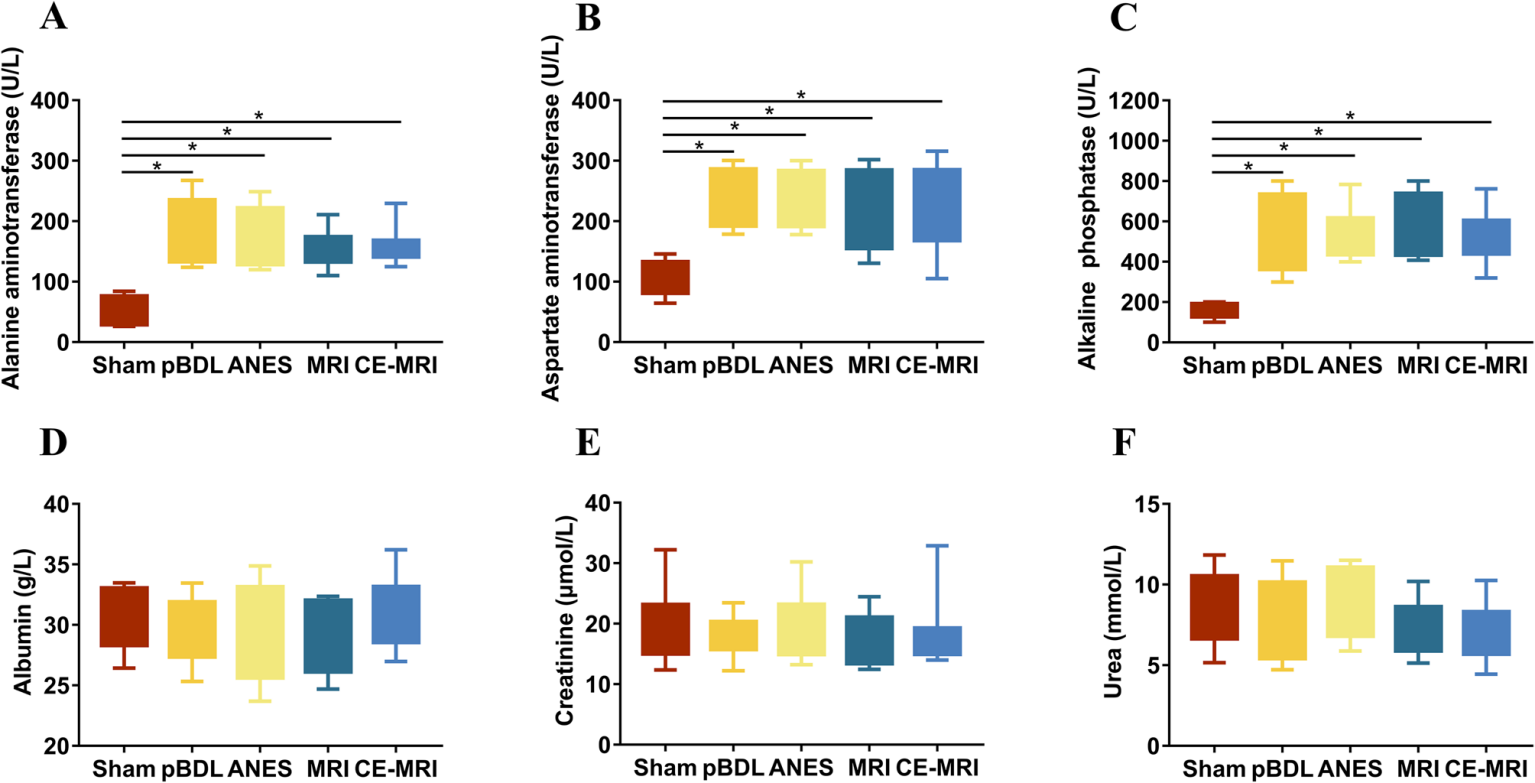
Biochemical data on week 6 in all groups. (**A**–**C**) Alanine aminotransferase, aspartate aminotransferase, and alkaline phosphatase levels were increased in the pBDL groups, compared with the sham group. These parameters in the repeated MRI and CE-MRI groups remained unchanged compared with the pBDL group. (**D**–**F**) Albumin, creatinine, and urea levels did not show significant increases after pBDL and repeated MRI examinations. Sham, only suture the abdominal wall. pBDL, partial bile duct ligation surgery alone; ANES, repeated anesthesia in the pBDL model; MRI, repeated magnetic resonance imaging under anesthesia in the pBDL model; CE-MRI, repeated contrast-enhanced magnetic resonance imaging under anesthesia in the pBDL model.

Although physiological changes may occur in mice because of emotional stimuli or potential physiological reactions after pBDL, physiological parameters (Fig. 2E,F) such as the heart rate and blood pressure did not show any alterations after pBDL. Liver damage, which was evaluated based on AST, ALT, and ALP levels, significantly differed between the sham and pBDL groups (P < 0.05), with the pBDL groups having significantly higher AST, ALT, and ALP levels than the sham group (Fig. 3A–C). However, the albumin level did not differ significantly between the sham and pBDL groups (Fig. 3D). No obvious changes in kidney function parameters, including the creatinine and urea levels (Fig. 3E,F), were observed among the groups.

### Impact of MRI on animal behavior, physiology, and biochemistry

The influence of MRI on animal behavior, physiology, and biochemistry was studied within the pBDL groups. Initially, there were no significant differences in animal motor behavior, balance, or physiological parameters when the ANES group was compared with the pBDL and sham groups (Figs. 2 and 3). Although ALT, AST, and ALP levels in the ANES group were increased, they were similar to those in the pBDL group, indicating that repeated anesthesia had no additional effect on the pBDL model. Next, in line with our results in the ANES group, the MRI group with high-decibel sequences revealed no differences in the results of the open-field test, rotarod performance, and physiological and biochemical parameters compared with those of the ANES and pBDL groups (Figs. 2 and 3). Representative images with high-decibel sequences are shown in Fig. S1.

### Pathological impact of the pBDL model and repeated MRI

**Figure 4:**
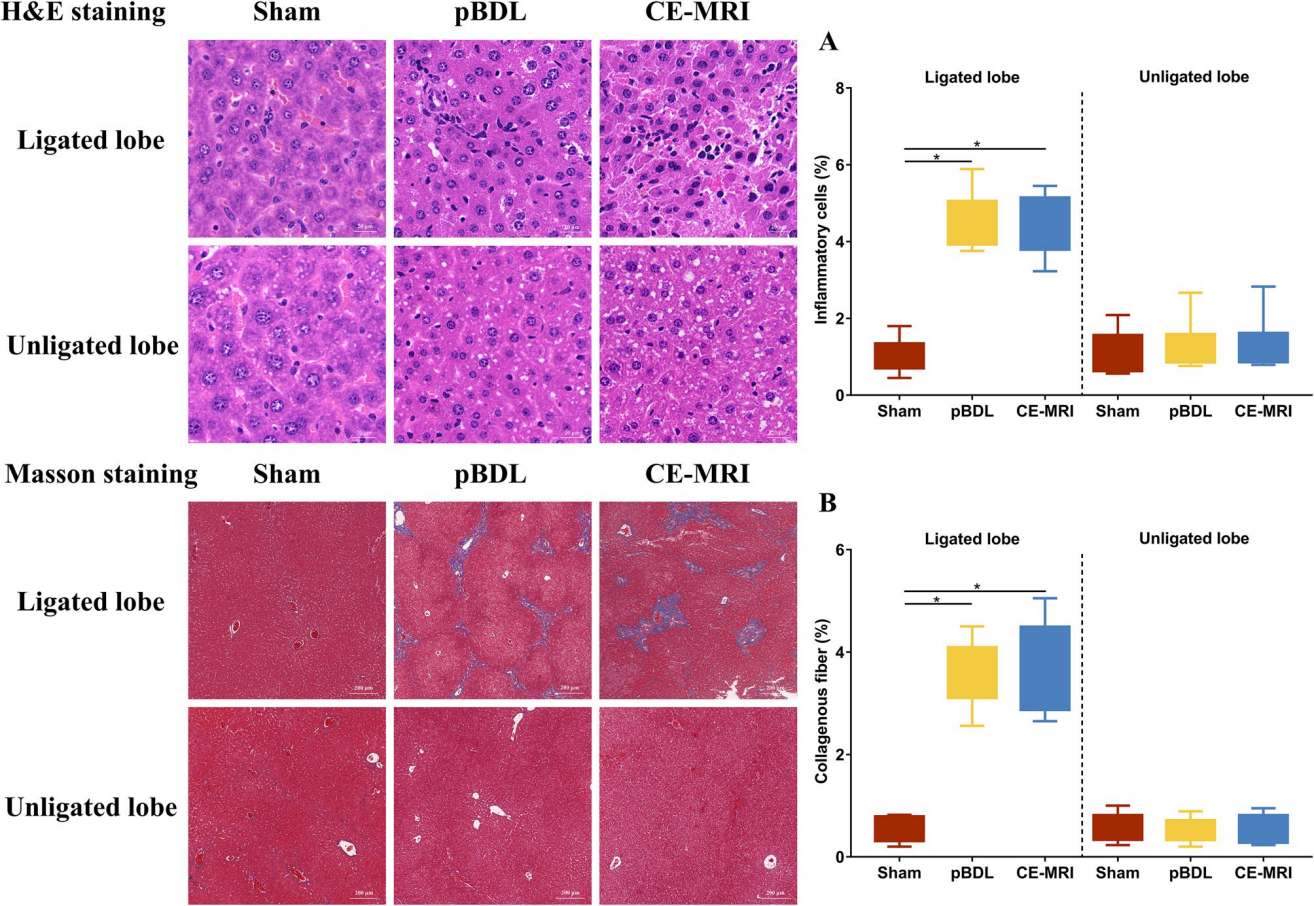
Lobe-specific changes in representative liver tissue from week 6 in the sham, pBDL, and CE-MRI groups comparing the unligated right lobe to the ligated left lobe in mice. (**A**) HE (original magnification, 400×) stain showing increased inflammatory cells (dark blue) in the ligated lobe of the pBDL and CE-MRI groups. (**B**) Masson stain (original magnification, 50×) showing significant fibrosis formation (collagen fibers, blue) in the ligated lobe of the pBDL and CE-MRI groups. HE, hematoxylin and eosin; Sham, only suture the abdominal wall. pBDL, partial bile duct ligation surgery alone; CE-MRI, repeated contrast-enhanced magnetic resonance imaging under anesthesia in the pBDL model.

The characteristic morphological alterations of the liver tissue that are induced by the pBDL were demonstrated in the standard HE staining (Fig. 4). Although the number of inflammatory cells in the pBDL and CE-MRI groups was greater than that in the sham group, the inflammatory cell counts in the CE-MRI group did not have notable changes compared with those in the pBDL group. In the unligated lobe, the number of inflammatory cells was similar in the sham and pBDL groups. Next, the formation of fibrosis was highly reliable with increased intrahepatic collagen deposition because of the ongoing fibrogenesis in the pBDL group. In the CE-MRI group, the formation rate of the collagenous fiber was not different compared with that in the pBDL group. Additionally, there were no increases in the collagenous fiber deposition in the unligated lobe.

## Discussion

In this study, we aimed to systematically understand the potential negative effects of t repeated MRI examinations in the pBDL model. These results indicated that although the pBDL model would result in minor liver damage, it did not impair locomotor performance or increase abnormal behaviors for at least 6 weeks after the surgery. As for the technical improvements for the pBDL model, first, we ligated the confluence of the left and middle bile ducts to avoid the anatomic variation of the branches of the bile duct, instead of ligating the left or middle bile duct^5^. Second, we used two surgical knots to tightly control the bile duct so that the complete obstruction of the bile duct^14^. Third, the location of the ligation was parallel to the right hepatic lobe at the transverse level, which was appropriate for MRI, as both the ligated and unligated lobes were visible on the same level. Fourth, the unligated liver lobe can compensate metabolically for the ligated left one. The adaptability of the liver is also known in humans; ligation of the left branch of the portal vein to stimulate the right lobe of the liver to grow is regularly done for preparing the liver for resection. Moreover, the results of the behavioral, physiological, and biochemical analyses showed that the outcomes of the pBDL model were consistent with the expected advantage of less tissue injury and fewer complications. Although the ALT, AST, and ALP levels at 6 weeks after the pBDL surgery were slightly increased in the pBDL groups, the overall levels in our study were lower than those previously reported^15,16^.

Moreover, the CE-MRI group did not have significant changes in their behavioral and physiological parameters, and the use of a contrast agent did not result in significant impairment in liver or kidney function compared with the MRI and sham groups, indicating that repeated MRI or CE-MRI showed good animal welfare in pBDL models. There is a general agreement among researchers that the number of experimental animals and their pain should be minimized. However, longitudinal studies are known to induce a higher burden on a single animal for a longer period^17^. Especially, echo planar imaging sequences, such as IVIM, DCE, and MRCP, which require fast switching rates and high gradient amplitudes, can lead to greater peripheral nerve stimulation and tissue heating than other sequences. To obtain sufficient spatial and temporal resolutions, MRI also requires a longer scan time and contrast agent injection^18^, causing additional impairments. In the ANES group, no behavioral, physiological, or biochemical changes were observed, indicating that repeated general anesthesia with proper concentration does not lead to these impairments in mice. Comparable findings have been reported for the impact on physiological changes after inhalational general anesthesia^19^, but that after intraperitoneal general anesthesia in mice had not been described yet. These encouraging results for the repeated MRI protocol are in line with the results in the mice in the pBDL groups. These results also corroborate the findings of a few previous works that showed no effects of MRI on behavior or locomotion in rats and mice^20,21^. But the molecular changes of intravenous anesthetics didn’t be investigated in our study. It might be noted based on the previous study which showed that ketamine had several effects on the immune system, for example, attenuation of natural killer cell activity or enhancing hypoxia-inducible factors^21^. Lastly, it is noteworthy that no changes in the analytical parameters were observed in the CE-MRI group with contrast-enhanced imaging. Taken together, all the results demonstrated that repeated contrast-enhanced MRI examination was well tolerated in pBDL mice. In future investigations, these findings gave us the foundation and have encouraged us to raise intriguing questions regarding the application of molecular MRI technologies in the pBDL model.

For fibrotic progression, inflammation has a critical role in the pathological process of liver fibrosis. Inflammatory cells are often recruited to the injured liver and act as initial responders to activate the signal pathway of fibrosis^22–24^. In our study, the level of the inflammatory cells at week 6 after pBDL remained high, which meant that injury-induced inflammation still existed and would continue to facilitate the progression of fibrosis^25^. Moreover, the level of inflammatory cells in the CE-MRI group was similar to those in the pBDL group, indicating that repeated MRI did not promote inflammation. Unlike the tissue samples in the BDL model, which may lead to liver necrosis but not form specific cholestatic liver tissue, the tissues in the pBDL model could form stable and homogeneous collagenous fibers, which are not affected by repeated MRI use.

There is a limitation in our study. Liver cirrhosis, a typical feature of chronic cholestasis, was not observed in the mice^26^. A possible explanation for these results may be the lack of an adequate observational period. Future studies on the current topic are therefore recommended.

In conclusion, we systematically investigated the potential long-term impairments of repeated MRI in pBDL models Based on our findings, we believe that pBDL is a reproducible model in scientific research. Furthermore, MRI, as a safe tool with many advantages on animal welfare for longitudinal evaluation, is well tolerated in mice with cholestasis. Therefore, the use of the pBDL model combined with repeated MRI has merit in the research of cholestatic fibrosis.

## Supplementary Information

Supplementary Information.

## Data Availability

The datasets generated during and/or analyzed during the current study are available from the corresponding author on reasonable request.
