# Supplementary material for: Impact of partial bile duct ligation with or without repeated magnetic resonance imaging examinations in mice
Source: Sci Rep. 2022 Dec 5;12:21014. doi: 10.1038/s41598-022-25318-8 (PMC9722823; doi:10.1038/s41598-022-25318-8)

Figure S1. Representative magnetic resonance images on week 6 after pBDL surgery at the transversal level of the liver showed that although our protocol had a higher decibel of noise and longer scan, the images could clearly show the difference between ligated and unligated lobes. T1WI, T1-weighted imaging; T2WI, T2-weighted imaging; IVIM, intravoxel incoherent motion imaging; MRCP, magnetic resonance cholangiopancreatography; DCE, dynamic contrast-enhanced imaging.


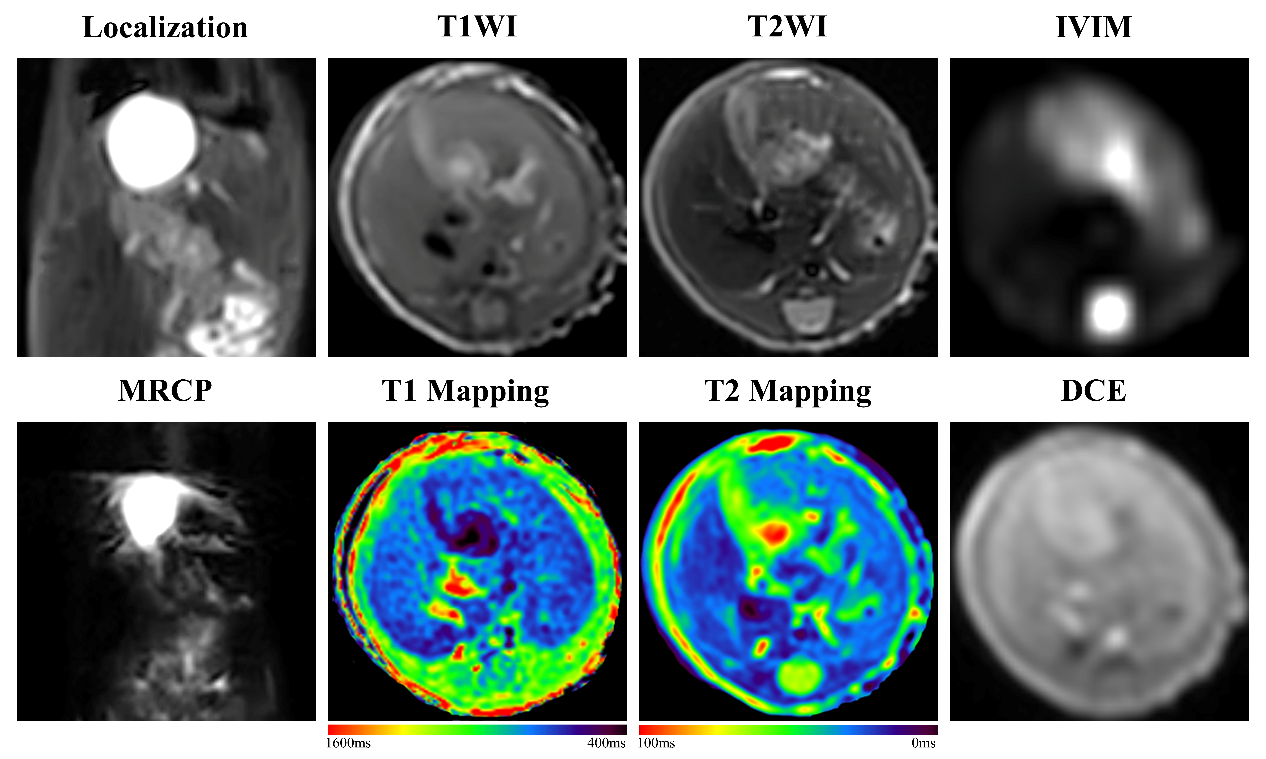

Supplement: Supplementary file 1 — Supplementary Information. [file 41598_2022_25318_MOESM1_ESM.docx]
